# MODY probability calculator for *GCK* and *HNF1A* screening in a multiethnic background population

**DOI:** 10.20945/2359-3997000000173

**Published:** 2019-09-25

**Authors:** Roberta Magalhães Tarantino, Gabriella de Medeiros Abreu, Ana Carolina Proença de Fonseca, Rosane Kupfer, Maria de Fátima Carvalho Pereira, Mario Campos, Lenita Zajdenverg, Melanie Rodacki

**Affiliations:** 1 Departamento de Medicina Interna Universidade Federal do Rio de Janeiro Rio de Janeiro RJ Brasil Seção de Diabetes e Nutrologia, Departamento de Medicina Interna, Universidade Federal do Rio de Janeiro (UFRJ), Rio de Janeiro, RJ, Brasil; 2 Laboratório de Genética Humana Instituto Oswaldo Cruz Fundação Oswaldo Cruz Rio de Janeiro RJ Brasil Laboratório de Genética Humana, Instituto Oswaldo Cruz, Fundação Oswaldo Cruz, Rio de Janeiro, RJ, Brasil; 3 Instituto Estadual de Diabetes e Endocrinologia do Rio de Janeiro Rio de Janeiro RJ Brasil Seção de Diabetes, Instituto Estadual de Diabetes e Endocrinologia do Rio de Janeiro, Rio de Janeiro, RJ, Brasil; 4 Departamento de Patologia Clínica Universidade Federal do Rio de Janeiro Rio de Janeiro RJ Brasil Departamento de Patologia Clínica da Universidade Federal do Rio de Janeiro, Rio de Janeiro, RJ, Brasil

**Keywords:** GCK, HNF1A, MODY, monogenic diabetes

## Abstract

**Objective:**

We aimed to identify the frequency of monogenic diabetes, which is poorly studied in multiethnic populations, due to GCK or HNF1A mutations in patients with suggestive clinical characteristics from the Brazilian population, as well as investigate if the MODY probability calculator (MPC) could help patients with their selection.

**Subjects and methods:**

Inclusion criteria were patients with DM diagnosed before 35 years; body mass index < 30 kg/m^2^; negative autoantibodies; and family history of DM in two or more generations. We sequenced HNF1A in 27 patients and GCK in seven subjects with asymptomatic mild fasting hyperglycemia. In addition, we calculated MODY probability with MPC.

**Results:**

We identified 11 mutations in 34 patients (32.3%). We found three novel mutations. In the GCK group, six cases had mutations (85.7%), and their MODY probability on MPC was higher than 50%. In the HNF1A group, five of 27 individuals had mutations (18.5%). The MPC was higher than 75% in 11 subjects (including all five cases with HNF1A mutations).

**Conclusion:**

Approximately one third of the studied patients have GCK or HNF1A mutations. Inclusion criteria included efficiency in detecting patients with GCK mutations but not for HNF1A mutations (< 20%). MPC was helpful in narrowing the number of candidates for HNF1A screening.

## INTRODUCTION

The frequency of monogenic diabetes mellitus (DM) has been underestimated in various populations (1). Most cases are caused by mutations in *GCK* [glucokinase gene] (GCK MODY [maturity onset diabetes of the young]) or *HNF1A* [hepatocyte nuclear factor 1-alpha gene] (HNF1A MODY) (2). Their molecular diagnosis is expensive but promotes the improvement of genetic counseling and treatment (3). Strategies to select the ideal subjects to screen for monogenic DM (MDM) in different populations are necessary to establish cost-effective diagnostic algorithms.

Different authors have developed clinical criteria for MODY screening based on age, family history and clinical characteristics (4,5). Although their use appears to be cost-effective (6), there is a concern that the screening based on clinical criteria would either miss part of the affected patients or still result in an excessive number of genetic tests (7). Therefore, authors have pursued optimal strategies for selecting patients. Shields and cols. developed a clinical prediction model that generates a probability of MODY (8) and shows good discrimination between MDM and type 1 (T1DM) or type 2 DM (T2DM) in European patients diagnosed under 35 years. The performance of this calculator in non-Caucasians is unknown.

The Brazilian population is very diverse and comprises individuals from multiple ethnic backgrounds, especially Caucasoid and Afro-descendants. There are scarce data about the prevalence of MDM and its optimal screening strategy in this setting. Our aim in this study was to estimate the frequency of MDM due to *GCK* or *HNF1A* mutations in patients with suggestive clinical characteristics and to investigate if the MODY probability calculator (MPC) could improve patient detection in this population (8,9).

## SUBJECTS AND METHODS

In this cross-sectional observational study, we selected patients clinically defined with monogenic diabetes from two specialized centers in Rio de Janeiro, between March 2012 and June 2015. MODY screening is not part of the routine laboratory panel of either center. We analyzed thirty-four unrelated probands from Brazilian families for mutations in *GCK* and *HNF1A.*

The inclusion criteria were age of DM diagnosis ≤ 35 years, body mass index (BMI) ≤ 30 kg/m^2^ or 95th percentile at onset, negative anti-glutamic decarboxylase antibody (anti-GAD) and anti-islet antigen 2 antibody (anti-IA2) antibodies and family history of diabetes in at least two generations, excluding the generation of the index case, and/or two or more first-degree relatives at the same side of the family. We excluded patients with T1DM; past diabetic ketoacidosis; clinical signs of insulin resistance (acanthosis nigricans, increased abdominal circumference and obesity); and secondary causes of diabetes.

The Ethics and Research Committee of the Clementino Fraga Filho University Hospital and State institute of Diabetes and Endocrinology of Rio de Janeiro approved this study protocol. We informed all participants about the aim of this study and provided verbal and written consent.

We calculated the positive predictive value (PPV) for MODY based on the MPC for each patient (8) and divided the patients into two groups. The *GCK* group included patients with fasting hyperglycemia (100-154 mg/dL); increased glycaemia after 75 g anhydrous dextrose <54 mg/dL and HbA1c < 7.5% (58 mmol/mol); and evolutionarily stable disease (even without antidiabetic drugs), most often asymptomatic and with hyperglycemia since birth (10). The *HNF1A* group included all other cases that met the inclusion criteria and that did not have the profile for the *GCK* group.

We isolated genomic DNA from peripheral blood leukocytes using QIAamp DNA Blood Mini Kit (Qiagen, Hilden, Germany). Also, we purified PCR products using Clean Sweep PCR Purification Reagent (Applied Biosystems, Vilnius, Lithuania). Then, we performed screening of the entire coding sequence of *GCK* and *HNF1A* genes through bidirectional Sanger sequencing using the Big Dye Terminator Kit v3.1 (Applied Biosystems, Austin, TX, USA), conducted on an ABI 3130 Automatic Genetic Analyzer (Applied Biosystems). Primers sequences are available upon request. We confirmed all mutations by bidirectional sequencing of a second PCR reaction. Then, we estimated the serum levels of anti-GAD and anti-IA2 antibodies by means of an enzyme-linked immunosorbent assay method (ELISA) using a EUROIMMUN kit.

We checked the variants identified against public Databases PubMed, Clinvar, dbSNP (https://www.ncbi.nlm.nih.gov/), Human Genome Mutation Database (HGMD^®^) (http://www.hgmd.cf.ac.uk/ac/), ExAC Browser (http://exac.broadinstitute.org), GnomAD (http://gnomad.broadinstitute.org/) and 1000 Genomes project database (http://www.internationalgenome.org/) to investigate their previous identification in the literature. We performed functional analyses using Mutation Taster (http://www.mutationtaster.org) (11-13).

We evaluated the differences between patients with mutations and others with Student’s t-test and chi-square tests. In addition, we performed statistical analysis using SPSS software (version 22.0).

## RESULTS

### Characteristics of the study group

We included 34 individuals (61.7% females) with a mean age of DM diagnosis and a duration of 19.8 ± 8.8 and 14.6 ± 9.9 years, respectively, as well as a mean BMI of 22.8 ± 3.2 kg/m^2^. 55.8% used insulin, and 41.2% used oral antidiabetic drugs (OAD). Their previous DM classifications included the following: non-classified in 38.2%, T1DM in 35.3%, T2DM in 23.5% and gestational diabetes (GDM) in 3%. Patients in the *GCK* (n = 7) and *HNF1A* (n = 27) groups had similar age at onset (16.1 ± 7.8 vs 20.8 ± 9.0 years; p = 0.286), diabetes duration (9.88.8 vs 15.4 ± 10.1 years; p = 0.82) or BMI (20.6 ± 4.0 vs 23.3 ± 2.8; p = 0.143).

None of the patients in the *GCK* group used insulin, and one used OAD. Most patients (70.3%) in the *HNFA* group used insulin (mean dose: 0.9 ± 0.4 UI/kg/day), and 48.1% used OAD.

### Genetic tests

We found MODY mutations (*GCK* or *HNF1A*) in 11 patients (32.3%). Fifty per cent of patients with non-classified DM had mutations in *GCK* or *HNF1A.* We fond mutations in 16.6% of those previously classified as T1DM and in none classified as T2DM. The only patient classified as GDM had a *GCK* mutation.

In the *GCK*-suspicious group, six cases had mutations (85.7%): Five were missense mutations: p.Tyr61Asp (c.181T>G; novel); p.Arg191Trp (c.571C>T); p.Thr228Met (c.683C>T); p.Ala384Val (c.1151C>T); p.Gly227Asp (c.680G>A); and one in-frame deletion p.Phe150del (c.449_451delTCT). We tested the patient in this group without the *GCK* mutation for *HNF1A,* and we found no mutations.

In the MODY-*HNF1A*-suspicious group, we found five mutations (5/27-18.5%); three missense substitutions: p.Gly31Asp (c.92G>A), p.Val133Glu (c.398T>A; novel) and p.Trp165Arg (c.493T>C); one nonsense mutation: p.Arg171Ter (c.511C>T); and one frameshift insertion: p.Thr433Hisfs*116 (c.1296_1297insC; (novel). Patients with *HNF1A* mutations used insulin less frequently than others. Those who used insulin reported a lower insulin dose/kg (Table 1). Other characteristics of those with or without *HNF1A* mutations are shown in Table 1.

**Table 1:** Clinical characteristics of patients according to mutations in the *HNF1A* gene

	Mutation		p

	Present (n = 5)	Absent (n = 22)	
Sex			
Female	1 (20%)	15 (68.2%)	0.048
Male	4 (80%)	7 (31.8%)	
Age of diagnosis (years)	17.6 ± 6.8	21.5 ± 9.4	0.237
BMI* (kg/m^2^)	22.08 ± 3.73	23.6 ± 2.6	0.154
Insulin use	1 (20%)	18 (81.8%)	0.006
OAD** use	3 (60%)	10 (45.4%)	0.557
Affected generations			
1	0	5 (22.7%)	0.054
2 or more	5 (100%)	17 (77.3%)	
Age of diagnosis/grade			
Childhood	0	3 (13.6%)	0.323
Adolescence	3 (60%)	6 (27.3%)	
Adult	2 (40%)	13 (59.1%)	
Diabetes presentation			0.583
Insidious	1 (20%)	3 (13.6%)	
Abrupt^#^	4 (80%)	19 (82.6%)	
Diabetes Duration (years)	8.6 ± 7.06	16.95 ± 10.13	0.432
MODY probability (PPV)	75.5 ± 0.0	35.7 ± 30.9	< 0.001
Mean insulin dose per kg of weight (units/kg)	0.37	0.9 ± 0.37	-

Note: Quantitative variables are presented as mean and standard deviation. Categorical variables are presented as total number (n) and percentage (%). The age groups used included the following: childhood, 0-9 years, adolescence, 10-19 years and adult, over 20 years (17).

* BMI: body mass index; ** OAD: oral anti diabetic drug; ^#^ Symptoms of insulinopenia such as polyuria, polydipsia and weight loss.

Among the 11 mutations, eight had already been described, and three were novel mutations. The mutation p.Tyr61Asp (c.181T>G), found in exon 2 of the *GCK* gene of one patient, is a missense mutation classified as pathogenic. The other two novel mutations occurred in the *HNF1A* gene (exons 2 and 6). We considered the missense mutation p.Val133Glu (c.398T>A) and the frameshift insertion p.Thr433Hisfs*116 (c.1296_1297insC) pathogenic because they alter the codon reading frame due to the insertion of a nucleotide (Table 2).

**Table 2:** Mutations identified in this study

Gene	Exon	Patient	Change in protein	Change in DNA	Consequence	SIFT prediction	SIFT score	Polyphen	Mutation Taster prob.	ClinVar	Reference
*GCK*	2	P40	p.Tyr61Asp	c.181T>G	Missense	Deleterious	0	Probably harmful	0.999999998323156	NA	This study, novel
	4	P1	p.Phe150del	c.449_451delTCT	In-frame deletion	NA	NA	NA	0.999999999980624	NA	Massa and cols., 2001
	5	P7	p.Arg191Trp	c.571C>T	Missense	Deleterious	0	Probably harmful	0.999999947468603	Pathogenic	Ellard and cols., 2000
	7	P29	p.Gly227Asp	c.680G>A	Missense	Deleterious	0	Probably damaging	0.999999999998095	NA	Domínguez-López and cols., 2013
	7	P9	p.Thr228Met	c.683C>T	Missense	Deleterious	0	Possibly harmful	0.99999999999911	Pathogenic	Stoffel and cols., 1992
	9	P32	p.Ala384Val	c.1151C>T	Missense	Tolerated	0.06	Possibly harmful	0.999999998673333	Uncertain meaning	Costantini and cols., 2014
*HNF1A*	1	P4	p.Gly31Asp	c.92G>A	Missense	Tolerated	0.29	Possibly harmful	0.995035768629034	Pathogenic	Chèvre and cols., 1998
	2	P28	p.Val133Glu	c.398T>A	Missense	Deleterious	0	Possibly harmful	0.999999999738075	NA	This study, novel
	2	P13	p.Trp165Arg	c.493T>C	Missense	Deleterious	0	Possibly harmful	0.999999998737327	NA	Tatsi and cols., 2013
	2	P37	p.Arg171Ter	c.511C>T	Nonsense	NA	NA	NA	1	Pathogenic	Vaxillaire and cols., 1999
	6	P5	p.Thr433Hisfs*116	c.1296_1297insC	Frameshift-insertion	NA	NA	NA	1	NA	This study, novel

NA: not applicable; prob: probability of causing disease.

**Figure 1:**
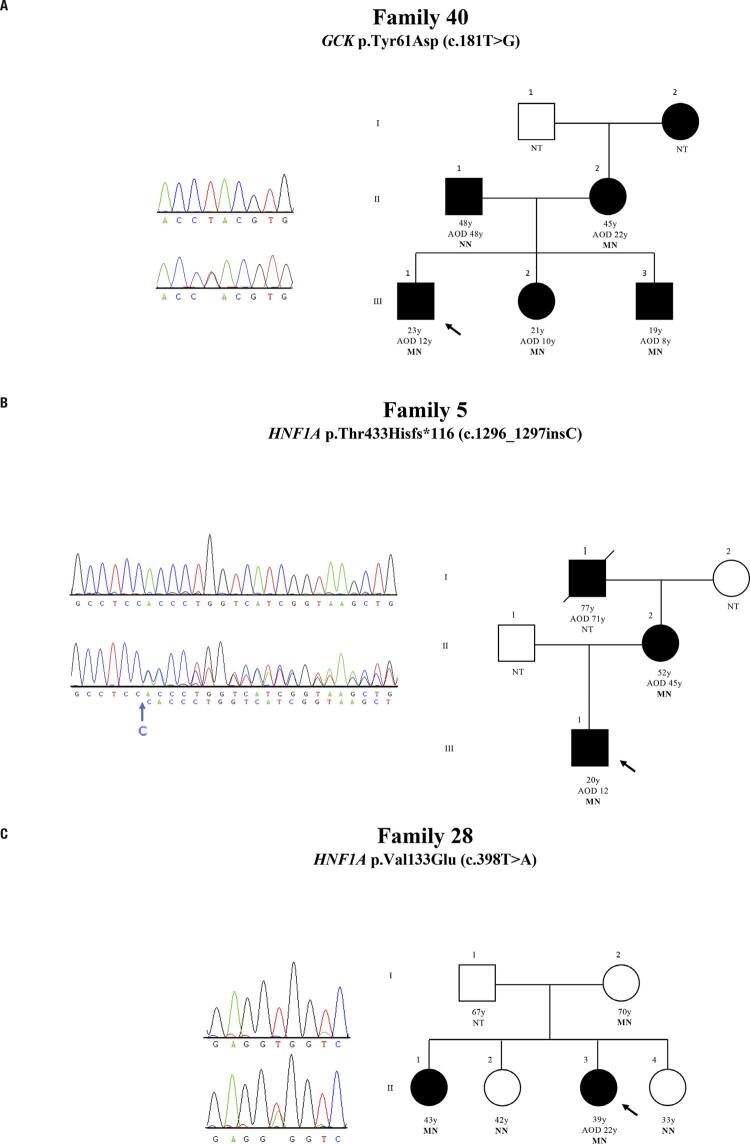
Pedigree and genotype of novel mutations. (A) Family 40 with the *GCK* p.Tyr61Asp (c.181T>G) in heterozygous state. K: allele T or G. (B) Family 5 showing the novel insertion HNF1A p.Thr433Hisfs*116 (c.1296_1297insC). The blue arrow indicates where the insertion occurs. (C) Family 28 presenting the *HNF1A* p.Val133Glu (c.398T>A). W: allele T or A. Filled symbols and empty symbols represent subjects with diabetes and healthy individuals, respectively. The present age of the individuals is show below the symbols in years, followed by age of diagnosis (AOD) in years and genotype interpretation. Genotypes are expressed by normal allele (N) and mutated allele (M); NT: Not tested. An arrow indicates the index case.

We recruited the family of all three probands with novel mutations, as presented in the pedigrees (Figure 1). We recruited four family members of the patient with the p.Tyr61Asp mutation in *GCK*. Then, we tested all four individuals with diabetes. The brother and sister had the same p.Tyr61Asp mutation, and we observed that it was inherited from the mother with diabetes. The father with recent onset DM (after 50 years of age) did not have the mutation. We recruited the mother with DM of the patient with the mutation p.Thr433Hisfs*116. She also had the same mutation. Four family members of the patient (three sisters and the mother) with the mutation p.Val133Glu in *HNF1A* were recruited. The only sister with diabetes also had the same mutation of the patient, and they inherited the mutation from their healthy mother. We believe it may be a case of incomplete penetrance uncommon with *HNF1A* mutations but already observed by other authors (14,15). In addition, the mutation p.Val133Glu was absent in the two healthy sisters.

### MODY probability calculator

In the sample as a whole, 61.8% of patients (n = 21) had PPV > 50%, and 50% (n = 17) had PPV > 75%, according to the MPC. In those with PPV > 50%, 47.6% had mutations, and in those with PPV > 75%, 52.9% had mutations.

### HNF1A group

The probability of MODY, according to MPC, was ≥ 50% in 14 patients (51.8%) and ≥ 75% in 11 patients (40.7%). All five patients in the *HNF1A* group with mutations had PPV for MODY ≥75% (Table 1). We found a mutation in 5/11 (45%) patients with PPV ≥75% and in 5/14 (35.71%) of those with PPV ≥ 50%.

### GCK group

Six patients had PPV ≥ 75%, and 1 had PPV between 50 and 75%. We detected *GCK* mutations in 5 patients with PPV ≥ 75% and in patients with PPV between 50 and 75%, but not in 1 individual with PPV ≥75%.

## DISCUSSION

In this study, we identified patients with phenotypes suggestive of MDM and performed mutation screening for *GCK* and *HNF1A* genes. We found mutations in 32.3%. Although a high frequency of mutations in these two genes have been reported in individuals with a clinical diagnosis of MDM from several populations, especially from Northern Europe (16,17), others (including Southern Europeans, Asians and Brazilians) have found a lower frequency of mutations, as true with our findings (18-21). In some populations, other types of diabetes might share clinical features with MDM more frequently than others. Alternatively, other genes implicated in the pathogenesis of MODY could be more frequent in these groups, such as *HNF4A*, insulin promoter factor-1 (*IPF-1)*, *HNF1B*, *NeuroD1* and others. In the other two studies with Brazilian cohorts (20,^21^), approximately 60% of patients with clinical suspicion of MDM did not have mutations in *HNF1A, GCK* or *HNF4A* genes, but we did not test other MODY genes.

In those with mild fasting hyperglycemia, we found *GCK* mutations in 85.7%, which suggests a high sensitivity for the clinical criteria. The frequency of *GCK* mutations in this study was slightly higher than previously reported for most populations (42.4 to 61%), including Brazilians (21-23). We found mutations in *HNF1A*, the most common gene for non-*GCK* MDM, in 18.5%, which was much like the data obtained by Santana and coworkers in the Brazilian population but lower than reported in other populations (16-19,24). Therefore, the clinical criteria for the selection of patients for *GCK* testing seem to be adequate in most populations, including ours. Surprisingly, we found the opposite for non-*GCK* MODY. This suggests that an improvement in the clinical criteria to adequately select patients for screening is necessary or that, alternatively, other genes should also be investigated.

We have evaluated if MPC (8) could help identify a group of patients that would be more suitable for screening. All patients with *HNF1A* mutations had PPV > 75%. If we had selected only subjects above this cutoff, 60% of the patients would not have been tested. This strategy would make the screening more cost-effective. However, the frequency of mutations in other genes in patients with a clinical diagnosis of non*-GCK* MODY is still not known for our population. It is possible that patients without *HNF1A* mutations could have alterations in other MODY genes, which would be overlooked with this strategy.

In this study, a cutoff for MODY probability of > 75% and >62%, based on MPC, was found in all patients with *HNF1A* and *GCK* mutations, respectively. Although in UK the current pick-up rate for MODY testing is PPV > 25% (9), other authors have found a good specificity and negative predictive value in higher cutoff values (> 62.5%) for detecting MODY in non-Caucasian population (25). Our findings suggest that higher cutoff values should be considered for MODY screening in non-Caucasian populations.

This study has some limitations. First, we included a limited number of patients. Secondly, we tested only two MODY genes for financial reasons. Additionally, we used only one methodology (Sanger sequencing) to investigate mutations. This method is unable to detect copy number variations, large deletions and duplications that can represent up to 3% of all genetic alterations of the *GCK, HNF1A* and *HNF4A* (26). Another concern is related to the absence of a control group. The selection of the studied population was done in a pragmatic way when the patient did not present clinical criteria of T1 or T2 DM. An additional potential limitation is the lack of C-peptide measurement as a screening tool for MODY. The strengths of our study included finding three novel mutations not previously reported and providing new information about the screening of monogenic diabetes in individuals with multiethnic backgrounds. For future studies, we aim to bypass these limitations and to perform functional genomic studies to confirm novel mutations as pathogenic ones.

In conclusion, we investigated MODY mutations in patients with clinical features suggestive of MDM from a multiethnic background. Approximately one third of patients with clinical features suggestive of MDM from a multiethnic background had *GCK* or *HNF1A* mutations. While clinical criteria were efficient for detecting patients with *GCK* mutations, we found *HNF1A* mutations in less than 20% of the cases. Although MPC has not been validated for non-Caucasians, its use as a screening tool for selecting patients to test for *HNF1A* mutations, using a cutoff of 75%, would reduce the number of tests in 60% and increase the percentage of positive cases to 45%. These data suggest that the use of the MPC could be a cost-effective strategy for selecting patients to screening for non-*GCK* MODY mutations, but it is important to consider the possible role of non-*HNF1A* mutations in non-Caucasian populations such as ours.
